# Protein expression in tension wood formation monitored at high tissue resolution in *Populus*

**DOI:** 10.1093/jxb/erx186

**Published:** 2017-06-13

**Authors:** Joakim Bygdell, Vaibhav Srivastava, Ogonna Obudulu, Manoj K Srivastava, Robert Nilsson, Björn Sundberg, Johan Trygg, Ewa J Mellerowicz, Gunnar Wingsle

**Affiliations:** 1 Department of Chemistry, Umeå University, Umeå, Sweden; 2 Computational life science cluster (CLiC), Umeå University, Sweden; 3 Division of Glycoscience, School of Biotechnology, Royal Institute of Technology, AlbaNova University Centre, Stockholm, Sweden; 4 Umeå Plant Science Centre, Department of Forest Genetics and Plant Physiology, Swedish University of Agricultural Sciences, Umeå, Sweden; 5 Crop Improvement Division, Indian Grassland and Fodder Research Institute, Jhansi, UP, India

**Keywords:** cell wall, cellulose, lignin, *Populus*, proteomics, tension wood, tissue resolution, xylogenesis

## Abstract

Tension wood (TW) is a specialized tissue with contractile properties that is formed by the vascular cambium in response to gravitational stimuli. We quantitatively analysed the proteomes of *Populus tremula* cambium and its xylem cell derivatives in stems forming normal wood (NW) and TW to reveal the mechanisms underlying TW formation. Phloem-, cambium-, and wood-forming tissues were sampled by tangential cryosectioning and pooled into nine independent samples. The proteomes of TW and NW samples were similar in the phloem and cambium samples, but diverged early during xylogenesis, demonstrating that reprogramming is an integral part of TW formation. For example, 14-3-3, reactive oxygen species, ribosomal and ATPase complex proteins were found to be up-regulated at early stages of xylem differentiation during TW formation. At later stages of xylem differentiation, proteins involved in the biosynthesis of cellulose and enzymes involved in the biosynthesis of rhamnogalacturonan-I, rhamnogalacturonan-II, arabinogalactan-II and fasciclin-like arabinogalactan proteins were up-regulated in TW. Surprisingly, two isoforms of exostosin family proteins with putative xylan xylosyl transferase function and several lignin biosynthesis proteins were also up-regulated, even though xylan and lignin are known to be less abundant in TW than in NW. These data provided new insight into the processes behind TW formation.

## Teaser

Proteome analysis of tissues at various stages of tension wood and normal wood formation revealed signaling and lignocellulosic proteins important for tension wood developmental processes in *Populus*.

## Introduction

Modeling variations in temporal and spatial protein expression in tissues of tree is essential for understanding their developmental processes and/or dynamic responses to external perturbations (Rantalainen *et al.*, 2008). Wood forms as a result of cell division in the vascular cambium. Cells produced in the vascular cambium differentiate to form either secondary phloem on the outer side of the cambium, which consists of conducting sieve elements connected to companion cells and non-conducting parenchyma cells as well as phloem fibers, or secondary xylem on the inner side of the cambium, made up of non-conducting parenchyma cells and xylem fibers along with conducting tracheary elements (Mellerowicz *et al.*, 2001). This process of xylem formation, or xylogenesis, is locally modified when a woody stem experiences pressure to bend or reinforce one side. In these situations, hardwood species form a specialized type of reaction wood called tension wood (TW), which, due to its specific structure and chemical composition, has contractile properties (Fisher and Stevenson, 1981; Mellerowicz and Gorshkova 2012; Fagerstedt *et al.*, 2014). In many species, included those from the genus *Populus*, TW formation is an example of the impressive reprogramming of wood biosynthesis that is triggered by a change of stem position in the gravitational field. This reprogramming involves stimulation of xylem cell formation at the TW stem side, formation of more fibers and fewer vessel elements, and alterations in fiber cell walls that confer contractile properties. In some, but not all, species these fibers form an additional gelatinous layer and are denoted G-fibers (Felten and Sundberg, 2013; Fagerstedt *et al.*, 2014; Groover, 2016).

The cell walls in normal wood (NW) fibers (S-fibers) usually contain three secondary wall layers (S1, S2, and S3), which are deposited over a primary wall layer and composed of cellulose microfibrils (approx. 50% of d.w.) and hemicelluloses (mostly glucuronoxylan, with small amounts of glucomannan) (Mellerowicz and Gorshkova, 2012). Each cell wall layer will later become impregnated with lignin. The walls of G-fibers have an additional G-layer, which typically replaces part of the S2 and the entire S3 layer. The composition of the G-layer varies greatly from the composition of the S-layers, since it contains more crystalline cellulose (approx. 80% of d.w.), which is organized into macrofibrils with a larger diameter and more axial orientation (Timell, 1969; Fagerstedt *et al.*, 2014), and a G-layer specific polysaccharide matrix (Mellerowicz and Gorshkova, 2012). The main matrix components of the G-layer are pectic galactans and type II arabinogalactans (Gorshkova *et al.*, 2015), and the minor components are mannans, xyloglucans, and sometimes a special form of xylan (Kim *et al.*, 2012). The G-fibers lignify at maturity, but the lignin is restricted to the middle lamella, primary wall layer (compound middle lamella) and S-layers, whereas the G-layer, which is the thickest layer of the cell wall, remains non-lignified (Pilate *et al.*, 2004; Fagerstedt *et al.*, 2014).

Tension wood has been the subject of multiple transcriptomic and a limited number of proteomic studies because it is an interesting example of the reprogramming that occurs during cellular differentiation and has interesting properties from the material point of view and as a substrate for saccharification. Most transcriptomic analyses have reported a decrease in transcripts related to lignin and xylan biosynthesis and an increase in the transcripts of the cellulose biosynthetic machinery and fasciclin-like arabinogalactan proteins (FLAs) during TW formation in comparison with NW or opposite wood (OW), e.g. the wood formed on the opposite side of the stem as TW (Dejardin *et al.*, 2004; Lafarguette *et al.*, 2004; Paux *et al.*, 2005; Andersson-Gunnerås *et al.*, 2006; Lu *et al.*, 2008; Jin *et al.*, 2011; Chen *et al.*, 2015). A more recent study, which applied the RNA-seq methodology to compare the transcriptomes of TW, OW and NW, reported significant transcriptome differences between the three types of wood, indicating that signaling during the stem tilting response concerns both sides of the stem (Chen *et al.*, 2015). A similar conclusion can be drawn from proteome and phosphoproteome analyses in poplar (Mauriat *et al.*, 2015). A time-course proteomic study identified 60 proteins that are differentially abundant within stem tissues during the tilting response (Azri *et al.*, 2009). To date, and to the best of our knowledge, no transcriptomic or proteomic analyses of TW responses in tissues representing defined developmental stages of TW and NW biosynthesis have been performed. Most studies have been based on wood samples containing pooled cells at different stages of differentiation, or samples of the entire stem. Such analyses cannot differentiate between specific developmental stages, which is necessary for deciphering the different processes that occur in cells along the developmental gradient. Here we present the quantitative global protein analysis of a series of samples of developing wood where each sample represents a specific differentiation phase, ending at the mature NW and TW fibers. This approach enabled us to analyse the differential expression of proteins at various stages of wood formation.

## Material and methods

### Plant material and protein extraction

**Fig. 1.**
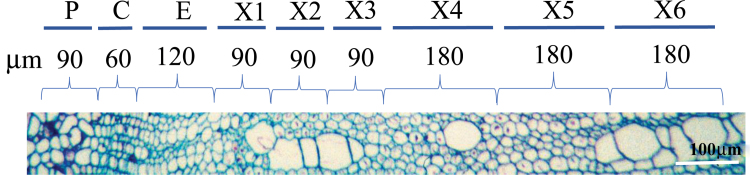
Transverse section through developing wood tissues from a representative tree illustrating the sampling strategy. Phloem (P), cambial zone (C), and expanding xylem (E) were represented by three, two, and four pooled sections, respectively. At the onset of lignification, three samples (X1–X3) with three pooled sections each were collected, followed by three samples (X4–X6) with six pooled sections each.

Field grown aspen (*Populus tremula* L.) trees were selected from a natural stand near Umeå, Sweden (63°50′N, 20°20′E). The trees were 5–6 m high and 3–4 cm in diameter at breast height. Tension wood was induced by bending and fixing the stems with strings, so that the midpoint of the stem was at an angle of about 45°. Bending was induced during the most active period of cambial growth. Upright trees were used as control. Stem pieces were collected from the midpoint of the stems. Samples were frozen in liquid nitrogen, transported to the lab on dry ice, and stored at −70 °C until processed. To prepare for tangential sections the sample was trimmed into 2 mm (tangential) × 10 mm (radial) × 15 mm vertical blocks consisting of phloem cambium and xylem. The blocks were cryo-sectioned at −20 °C with an HM 505E microtome (Microm labogeräte, Walldorf, Germany) according to Uggla and Sundberg (2002). Sections with a thickness of 30 µm were obtained from phloem, across the cambium and into the xylem. The radial position of the tangential sections was determined in cross-section samples taken at regular intervals during tangential sectioning. The sections were then pooled to represent tissues from developmental stages as described in Fig. 1. Phloem (P), cambial zone (C), expanding xylem cells (E) were represented by one pooled sample each, whereas lignifying and maturing xylem was represented by six pooled samples (X1–X6).

Tissues from the pooled samples were ground in a mixer-mill (MM 301, Retsch GmbH, Germany) for 1 min, after which highly water-soluble proteins were extracted as described in Bylesjö *et al.* (2009), based on a method presented by Giavalisco *et al.* (2003). Briefly, protease inhibitor (Roche complete, Sigma-Aldrich, Darmstadt, Germany) was added to 10 ml of extraction buffer (100 mM KCl, 20% glycerol, 50 mM Tris, pH 8.0). Tissue powder was then dissolved in 100 μl of the buffer and left for 10 min at 4 °C. The homogenate was centrifuged for 30 min at 226 000 *g* and 4 °C. The top 80 μl of supernatant was collected in 0.5 ml PCR tubes. The extracted proteins were then reduced by adding 5 μl of a DTT solution to a final concentration of 20 mM and incubated for 10 min at 95 °C using a thermocycler. The tubes were then transferred to ice and 10 μl of iodoacetamide solution was added to a final concentration of 80 mM, after which the samples were incubated for 20 min at room temperature in the dark to allow alkylation. The samples, diluted to 200 μl, were then applied to a pre-wetted membrane (MultiScreen Filter Plate with Ultracel-10 Membrane, Millipore MAUF01010) and centrifuged for 60 min at 2000 *g* and 25 °C (Centrifuge Heraeus Multifuge 3 S-R, rotor 75006444, Thermo Fisher Scientific, Waltham, MA, USA). The samples were washed twice with 200 μl of 0.2 M ammonium bicarbonate before 50 μl of sequencing-grade modified trypsin (5 ng/μl) (Promega, Madison, WI, USA) was added for overnight digestion. Peptides were eluted onto a collection plate by three repeated centrifugations using 40 μl of 0.2 M ammonium bicarbonate. Samples were then evaporated until dryness, dissolved in 25 μl of 0.1% formic acid and stored at −80 °C until use. The pellet that remained from each sample after the extraction of soluble proteins was treated the same way as the soluble proteins, with the addition that another 50 μl of trypsin was added after the overnight digestion, after which the pellet was left for another 4 h before the peptides were extracted.

### Proteome analysis and protein identification and quantification

The proteins identified from the soluble and pellet fractions were combined for each sample prior to final analysis. Proteome analysis and protein identification and quantification were performed using mass spectrometry as described by Obudulu *et al.* (2016). In essence, the peptides were separated using a nanoACQUITYTM ultra-performance liquid chromatography system (Waters, Milford, MA). Two microliters of each sample was loaded onto a PepMap100, nanoViper Acclaim® C18 trap column (100 μm i.d. × 2 cm, 5 μm particles, 100 Å pores; Thermo Scientific). The samples were then eluted from the trap column and separated on an HSS T3 (High Strength Silica T3) C18 analytical column (75 μm i.d. × 200 mm, 1.8 μm particles; Waters, Milford, MA, USA), using a linear 80-min gradient of 1–40% solvent B (3:1 ACN/2-propanol) balanced with 0.1% aqueous formic acid (solvent A) at a flow rate of 300 nl min^−1^. The eluate was passed to a Waters Synapt G2 HDMS mass spectrometer equipped with a nanoflow electrospray ionization interface operating in positive ionization mode with a minimal resolution of 20 000. All data were collected in continuum mode and mass-corrected using Glu-fibrinopeptide B and leucine enkephalin as reference peptides.

Proteins were classified as occurring in the sample if at least one of the peptides was sequence-unique (Silva *et al.*, 2005; Distler *et al.*, 2014). In total, 4675 unique peptides corresponding to 1050 proteins were quantified. Differences between zones in the wood series were subsequently investigated in detail using orthogonal projections to latent structures (OPLS) and OPLS discriminant analysis (OPLS-DA) models (Trygg and Wold, 2002, 2003; Rantalainen *et al.*, 2008).

### Pairwise correlation

Pairwise models were created to investigate the spatial progression from cambial initials to mature phloem and xylem, as well as to reflect chronological developmental sequences. Prior to modeling, the datasets were column-centered without scaling. An initial principal component analysis (PCA) model for all of the samples and zones provided a global overview. Next, OPLS-DA was used to model and identify the pairwise relationships between NW and TW within the zones. Original definitions, model statistics, selection criteria and detailed descriptions of the PCA, OPLS and its discriminant analysis variant are presented in Trygg and Wold (2002, 2003) and Rantalainen *et al.* (2008).

Significance testing of proteins (the significance of changes in abundance of proteins in the TW *versus* the NW, and their association with specific developmental stages/relationships) was performed by calculating jack-knife confidence intervals, with α=0.05 set as the significance limit (Efron and Gong, 1983; Wiklund *et al.*, 2008; Wold, 1978). Furthermore, the result of the OPLS was re-examined by means of a univariate analysis consisting of multiple pairwise comparisons to provide additional data analysis for a comprehensive and robust biological interpretation. The averages of all replicates from the TW and NW data (pairwise analysis) in each zone were used for fold change calculations. A fold change value >1.5 was considered biologically relevant. All univariate statistical analyses, including the calculation of sample average, mean, and fold change values were performed in Microsoft Excel. The results for the differentially expressed proteins obtained from the multivariate OPLS statistics were compared with the results for proteins from the univariate statistics with fold change values greater than 1.5 to increase the significance and consistency of the results (Saccenti *et al.*, 2014; Shiryaeva *et al.*, 2012). Lists of all the proteins detected and all the differentially expressed proteins can be found in the Supplementary Dataset S1 at *JXB* online.

### Pathway analysis

Pathways associated with the significantly differentiated proteins along the wood developmental series were examined using information obtained from the Kyoto Encyclopedia of Genes and Genomes (KEGG) database (Kanehisa and Goto, 2000; Kanehisa *et al.*, 2012) and MAPMAN, a user-driven tool providing pathway and biological process information (Thimm *et al.*, 2004). The expression patterns across the series of wood development stages were visualized using PermutMatrix software v.1.9.3 (Caraux and Pinloche, 2005). These resources identify the molecular processes that are most affected by expression differences and have been shown to efficiently link the functions of proteins to biological pathways (Hucka and Le Novère, 2010; Srivastava *et al.*, 2013).

The mass spectrometry proteomics data have been deposited at the ProteomeXchange Consortium via the PRIDE (Vizcaíno *et al.*, 2016) partner repository with the dataset identifier PXD005715.

## Results and discussion

We used tangential sections of upright and tilted stems (Fig. 1) to characterize the proteome of NW and TW along the developmental gradient. Overall, nine different developmental stages were sampled, covering a radial distance of approximately 1 mm. Among the detected peptides, we focused on those that were unambiguously mapped to proteins in order to examine the possibility that certain protein isoforms may be differentially expressed. We have also indicated all peptides that were found to be shared between two or more proteins (see e.g. Supplementary Fig. S1 and Supplementary Dataset S1).

**Fig 2.**
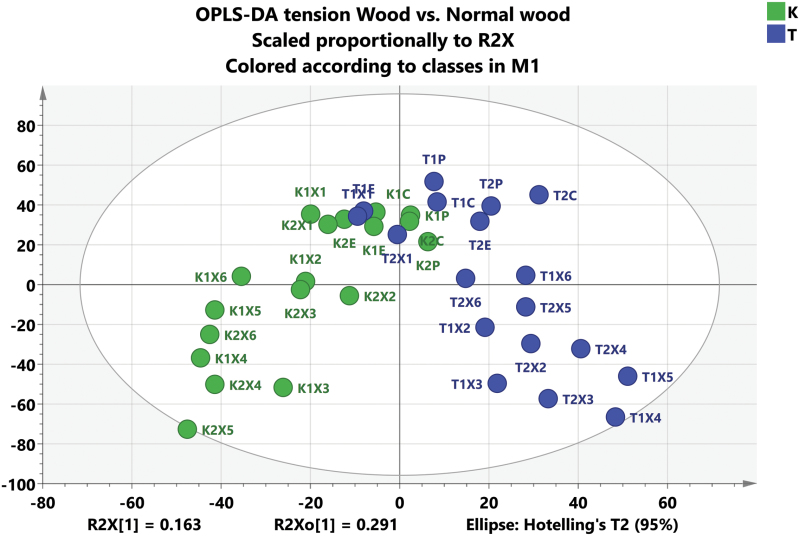
Score scatter plot of peptides from an OPLS-DA analysis of extracted fractions. Soluble and insoluble proteins were combined in the analysis. Samples were collected from successive tangential sections of the *Populus* stem and pooled into cambium (C), phloem (P), xylem expansion zone (E), and subsequent xylem cell differentiation (X1–X6). Green, normal wood (two separate trees); blue, tension wood (two separate trees).

**Fig. 3.**
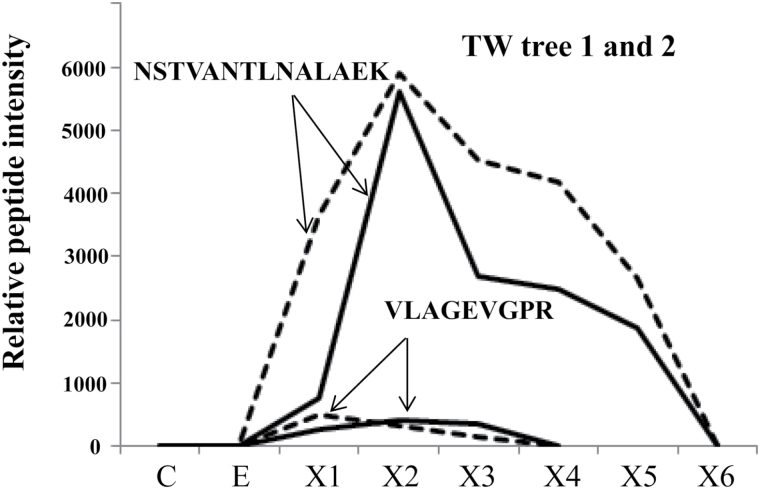
Expression profiles of unique peptides from two isoforms of ACC oxidase from samples of developing tension wood (TW) in two trees of *Populus*. The peptides, NSTVANTLNALAEK (unique in Potri.002G078600, *Pt*xtACO1) and VLAGEVGPR (unique in Potri.017G135800), were quantified in samples covering wood developmental series (see Fig. 1). Tree 1, solid line; tree 2, dotted line. These peptides were not detected in the normal wood trees.

To investigate protein expression in TW and NW, the intensities of uniquely mapped peptide markers were normalized and used for the OPLS-DA analysis (Fig. 2). The OPLS-DA analyses showed that the proteomes of developing TW and NW are initially quite similar, but the protein expression patterns between NW and TW begin to diverge as the xylem tissues differentiate. We selected two isoforms of ACC oxidase, one of which (ACO1) has been shown to be up-regulated during TW formation (Andersson-Gunnerås *et al.*, 2003, 2006), to exemplify the protein expression differences between TW and NW tissues. Both of these isoforms were highly abundant in the X2 and X3 zones of TW, but were not expressed in the corresponding zones of NW (Fig. 3); this indicates that the X2 zone of TW is already metabolically altered when compared with the same zone of NW. The protein with the highest overexpression in TW, based on fold change measurements of all proteins examined in our analysis, was ACO1, which is consistent with results reported by Mauriat *et al.* (2015).

### Pairwise comparison of the phloem, cambium, expansion zone, and xylem

We employed pairwise modeling to identify proteins that were differentially expressed at successive stages of wood formation in the TW and NW samples. OPLS-DA models were created for each pairwise relationship along the wood series. All of the OPLS-DA models were significant according to cross-validation by jack-knifing, and the Q2 value was used to measure the predictive robustness of each model. The corresponding differentially expressed peptides obtained from each zone are listed in Supplementary Dataset S1.

**Fig. 4.**
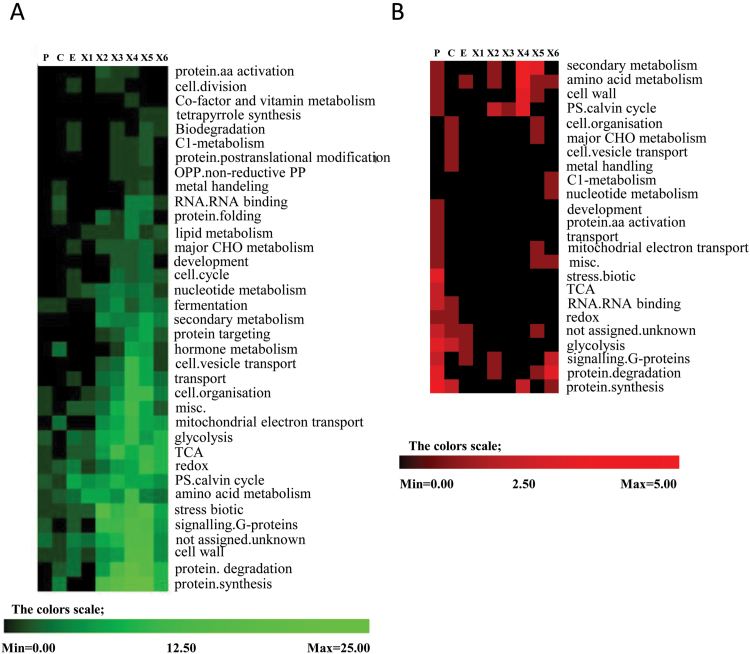
Number of differentially expressed proteins of *Populus* in tension wood (TW) as compared with normal wood, per developmental stage samples based on pairwise comparisons, and their functional classification according to Mapman. (A) Distribution of up-regulated proteins in TW within the annotated functional categories. (B) Distribution of down-regulated proteins in TW within the annotated functional categories. Samples designation as in Fig. 1. The number of differential proteins was based on unique peptide quantification.

An overall picture of the protein alterations between TW and NW, based on the OPLS-DA pairwise analyses, is presented in Fig. 4. Many functional categories (according to Mapmann classification) were found to be more abundant in TW xylem zones X1–X6 than in the corresponding zones of NW, indicating increased metabolic activity in TW at these developmental stages. In particular, the protein degradation and biosynthesis, biotic stress response and redox, cell wall biosynthesis, and signaling categories were highly up-regulated (Fig. 4A). We also performed an enrichment analysis using REVIGO (Supek *et al.*, 2011), which utilizes hierarchical presentations of non-redundant GO terms to facilitate interpretation, to reveal the metabolic processes that differed between TW and NW (Supplementary Dataset S2). A major fraction of the protein biosynthesis group belonged to ribosomal proteins (Supplementary Dataset 1). The protein degradation category comprised the proteasome complex, ubiquitin, peptidases and proteinases. A sharp up-regulation of these groups in the X2 zone of the TW tissue (Fig. 4A) reveals that X2 is the phase when cellular metabolism is most intensively reprogrammed in TW, possibly corresponding to G-layer initiation. The REVIGO enrichment analysis for TW showed an abundance of glucose and hexose catabolic processes in the X2 and X3 zones, which then shifted towards the up-regulation of proteins involved in the catabolism of purine-containing compounds in the X4 and X5 zones (Supplementary Dataset S2). Two end-products of complete purine degradation, glyoxylate and ammonia, have been proposed to be recycled for the synthesis of organic molecules that can be utilized for new growth (Werner and Witte, 2011). Different classes of proteins were also up-regulated in the X1 zone of TW, a phase where no G-layer structure is thought to be formed.

Interestingly, differences in metabolic activity were also found in the phloem of TW and NW samples. The TW phloem, when compared with the NW samples, revealed a down-regulation of many protein classes, such as glycolysis, TCA cycle, biotic stress, and protein metabolism (Fig. 4B). This suggests that general cellular metabolic activity shifts from phloem to xylem during TW biosynthesis.

### Secondary messenger proteins and regulatory proteins

The physiological and molecular signals that induce TW formation remain unknown. Table 1 lists the signaling proteins that were detected at higher levels in the X2 zone of TW than in the X2 zone of NW. There were clear metabolic differences between the wood types, a finding that is particularly interesting for detecting the proteins that play an important role in TW development. Calcium (Ca^2+^) is an important and ubiquitous secondary messenger in cells (Toyota and Gilroy, 2013), as is calreticulin, a Ca^2+^ storage protein (Azri *et al.*, 2009). We found that calreticulin b proteins and the calcium-binding EF-hand protein were elevated in the TW X2 zone. The EF-hand motif is the most common calcium-binding motif found in proteins. This supports the idea that the Ca^2+^ ion participates in TW induction via various pathways.

**Table 1.** *Signaling and mitochondrial proteins detected at higher levels in the xylem X2 zone of* Populus tremula *tension wood when compared with the corresponding zone of normal wood*

Protein	Function	MapMan biological process
Potri.002G099800, PtGRF1/2/4a	General regulatory factor 2	Signaling 14-3-3 proteins
Potri.005G162400, PtGRF1/2/4b	General regulatory factor 2	
Potri.017G113300, PtGRF3/5/7b	General regulatory factor 7	
Potri.004G101700, PtGRF3/5/7a	General regulatory factor 7	
Potri.005G157700, PtGRF6/8b	General regulatory factor 8	
Potri.002G103800, PtGRF6/8a	General regulatory factor 8	
Potri.001G117900, similar AT1G12310	Calcium-binding EF-hand family protein	Signaling calcium
Potri.013G009500, similar AT1G09210	Calreticulin 1b	
Potri.008G126600, similar AT5G08680	ATP synthase α/β family protein	Mitochondrial electron transport
Potri.010G116600, similar AT5G08680	ATP synthase α/β family protein	
Potri.005G166000, similar ATMG01190	ATPase, F1 complex, α subunit protein	
Potri.004G177500, similar AT4G38510	ATPase, V1 complex, subunit B protein	
Potri.001G028000, similar AT3G27890	NADPH:quinone oxidoreductase	
Potri.015G041600, similar AT1G48630, RACK1B_AT	Transducin/WD40 repeat-like superfamily protein	Signaling G-proteins
Potri.017G001200, similar AT3G59920, ATGDI2	Guanosine nucleotide diphosphate dissociation inhibitor 1	

Another protein family, the 14-3-3 proteins, showed elevated expression in the TW X2 zone. In plants, 14-3-3 proteins are encoded by a large multigene family, of which 14 genes have been identified in *Populus* (Tian *et al.*, 2015). They are involved in signaling pathways that regulate plant development and protection from stress (Darling *et al.*, 2005). Six of these genes have shown high transcript abundance in differentiating xylem and basal stem undergoing secondary growth in *Populus* (http.///www.popgenie.org/, Tian *et al.*, 2015). The 14-3-3 isoform has been identified as being part of the protein–G-box complex and is therefore named general regulatory factor (GRF) (DeLille *et al.*, 2001). These isoforms regulate the activities of a wide array of target proteins via protein–protein interactions, which involve binding to pSer/pThr residues of the target protein (Li and Dhaubhadel, 2011). Our proteomic analysis identified and quantified several 14-3-3 proteins similar to those described by Tian *et al.* (2015). The finding that several of the six paralogous 14-3-3 proteins were induced in TW tissue suggests that these proteins may have a key role in the development of *Populus* TW.

One of the most important regulators of signal transduction in plants is the Rac/Rop family, as members of this family participate in pathways that influence the growth and development of plants, along with adaptation to environmental conditions (Berken, 2006, Kawano *et al.*, 2014). We found elevated concentrations of various GTPases in TW tissue, demonstrating the significance of Rac/Rop signaling in TW development (Table 1, Supplementary Fig. S1). This category of GTPase regulators has rarely been discussed in relation to TW and was just recently described in a phosphoproteome study (Mauriat *et al.*, 2015).

Reactive oxygen species (ROS) can act as secondary messengers, and are essential for auxin-induced gravitropic signaling in maize roots (Joo *et al.*, 2001). Furthermore, Azri *et al.* (2013) suggested that a thioredoxin *h* (TRXh) isoform participates in signal transduction during TW formation in inclined poplar stems. Like Azri *et al.* (2013), we found one thioredoxin *h* protein, and other forms of TRXs, to be elevated in TW compared with NW (see Supplementary Fig. S1).

Furthermore, we found higher protein levels for paralogs of PDI-like 1–2 and PDI-like 1–4 in the TW X zones than the NW X zones. PDI contains TRX domains and catalyses disulfide bond formation in the oxidizing environment of the endoplasmic reticulum, working to stabilize the tertiary and quaternary structures that arise during protein folding (Gupta and Tuteja, 2011). Increased levels of various forms of superoxide dismutases, ascorbate peroxidases and glutaredoxins in the TW X2–X6 zones provides further evidence for increased oxidative stress during TW development (see Supplementary Fig. S1). These types of proteins not only protect organisms against the toxic effects of ROS, but also regulate intracellular signal transduction (Foyer and Noctor, 2011; Yu *et al.*, 2011). Intermediates of purine degradation have been proposed to protect plants from ROS (Werner and Witte, 2011). Furthermore, it has been shown that ROS regulate cell growth by activating Ca^2+^ channels (Foreman *et al.*, 2003). Overall, this supports the hypothesis that ROS participate as secondary messengers during TW development.

A few transcriptional regulators (TR) and factors (TF), namely TUDUR-SN1, (Potri.015G109300), GATA type zinc finger transcription factor family protein (Potri.009G087200) and two NmrA-like negative transcriptional regulator family proteins (Potri.005G228700 and Potri.002G034400), showed elevated expression in the TW X zones (Supplementary Dataset S1). TUDOR-SN1 in Arabidopsis has been shown to have a function in mRNA catabolism during stress as a positive regulator of mRNA decapping (Gutierrez-Beltran *et al.*, 2015). The GATA and NmrA proteins have been implicated, among others, in nitrogen signaling and regulation (Behringer and Schwechheimer 2015). A GATA-type zinc finger TF family protein (PU06749) was one of the highest expressed TF genes in the developing xylem region of TW (Andersson-Gunnerås *et al.*, 2006).

### Energy-related proteins

Ribosomal proteins (r-proteins) were highly up-regulated in TW xylem zones (Supplementary Dataset S1). The 68 r-proteins were found at elevated levels in the TW tissue, with 16 of them already at elevated levels in the X2 zone. One possible explanation for this finding is that r-proteins are among the most abundant proteins within the cell and are therefore more easily detected with mass spectrometry techniques. However, our results indicate a substantial induction of r-proteins in TW, a finding that was supported by the REVIGO enrichment analysis (Supplementary Dataset S2). This suggests the synthesis of new proteins during TW development. The up-regulation of r-protein biogenesis in TW supports the hypothesis that the xylem cellular metabolism is reprogrammed during TW development.

Ribosome biogenesis and mRNA translation are energy-demanding processes, as is the polymerization of cell wall components. Hence, we found that many of the mitochondrial electron transport proteins, particularly those involved in the ATP complex, were up-regulated in TW compared with NW (Table 1; Supplementary Dataset S1). Several of the ATPase complex proteins already showed elevated levels in the X2 zone of TW (Table 1). This preparation for high energy demand further supports the hypothesis of cellular reprogramming during TW development.

### Cell wall carbohydrate biosynthesis

**Fig. 5.**
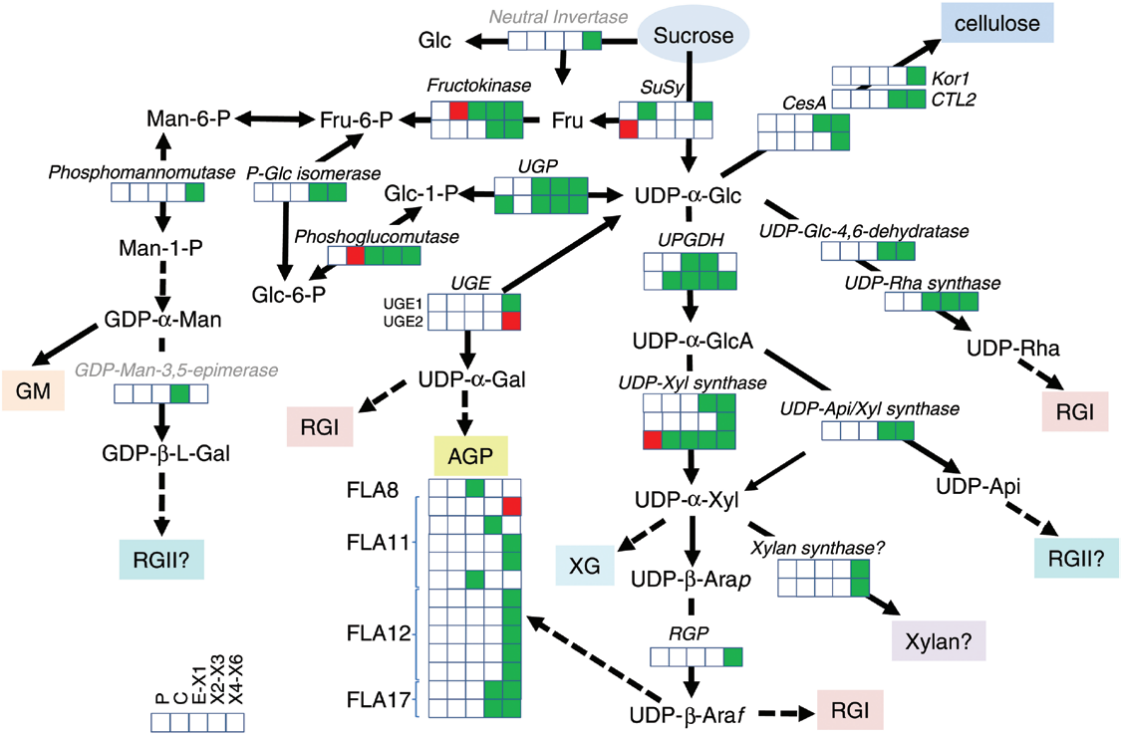
Differentially expressed enzymes in pathways leading to the biosynthesis of cell wall carbohydrate polymers at different stages of tension wood development, as compared with normal wood formation. Green, up-regulation; red, down-regulation. The proteins include the following: two isoforms of UGP (Potri.004G074600 and Potri.017G144700), fructokinase *Pt*FRK2B (Potri.007G129700), phosphoglucomutase (Potri.008G132500), sucrose synthases *Pt*SUS7 (Potri.017G139100) and *Pt*SUS3 (Potri.002G202300), cellulose synthases *Pt*CesA4 (Potri.002G257900) and *Pt*CesA8-B (Potri.004G059600), poplar korrigan *Pt*Cel9A1 (Potri.003G151700), chitinase-like protein *Pt*GH19A (Potri.010G141600), UDP-Glc-6-dehydrogenase (UGDH; Potri.017G237200 and Potri004G118600), UDP-D-glucose/UDP-D-galactose 4 epimerase (UGE) isoforms *Pt*UGE1 (Potri.003G123700) and *Pt*UGE2 (Potri.001G090700), UDP-Glc-4,6-dehydratase (Potri.001G383500), UDP-Rha synthase (Potri.003G120000), UDP-Xyl synthases (UXS) *Pt*UXS3 (Potri.001G237200), *Pt*UXS6 (Potri.008G053100) and *Pt*UXS7 (Potri.010G207200), fasciclin-like arabinogalactan proteins *Pt*FLA8 (Potri.014G071700), five isoforms similar to *At*FLA11 (Potri.012G127900, Potri.015G129400, Potri.012G015000, Potri.009G012100, Potri.006G129200), five isoforms similar to *AtFLA12* (Potri.013G151400, Potri.015G013300, Potri.004G210600, Potri.009G012200, Potri.013G014200), and two isoforms similar to *At*FLA17 (Potri.008G012400 and Potri.010G244900), xylan synthase *At*IRX10 homologs *Pt*GT47A1 and 2 (Potri.001G068100 and Potri.003G162000), UDP-API/UDP-XYL synthase 1 (Potri.009G150600), 3-deoxy-D-manno-octulosonate-8-phosphate synthase (KDO-8-phosphate synthase, Potri.002G061900), and GDP-Man-3,5-epimerase (Potri.T103900).

In developing xylem cells, UDP-Glc is directly used for the biosynthesis of cellulose and indirectly used, after conversion to various nucleotide-sugars, for the biosynthesis of all other cell wall polysaccharides. We found that a majority of key enzymes involved in UDP-Glc metabolism were more abundant in developing TW than in NW, especially at the later stages of xylogenesis (X4–X6) (Fig. 5), corroborating previous conclusions on TW metabolism reprograming based on transcriptome abundance (Andersson-Gunnerås *et al.*, 2006). Similarly, uridine monophosphate kinase, which synthesizes UDP, was highly expressed in TW zone X5. Some other enzymes involved in general sugar activation, such as two isoforms of UDP-Glc pyrophosphorylase (UGP), fructokinase (*Pt*FRK2B, Roach *et al.*, 2012), and phosphoglucomutase, were also up-regulated in the developing xylem, mirroring their transcript behavior (Andersson-Gunnerås *et al.*, 2006), but they were down-regulated in the cambium. This indicates an increased flux of sugars to the TW for cell wall biosynthesis, which might occur at the expense of sugar consumption in the cambium. Interestingly, one sucrose synthase (SUS) isoform, *Pt*SUS7 (An *et al.*, 2014), was down-regulated in the phloem, and another isoform, *Pt*SUS3, was up-regulated in the xylem (Fig. 5 and Supplementary Dataset S3). *Pt*SUS7 is a member of a clade of SUS proteins that are highly expressed in the phloem of stems producing NW (http://aspwood.popgenie.org, Sundell *et al.*, 2016), whereas *Pt*SUS3 belongs to a separate clade, which is different from both the phloem-abundant and the wood-abundant isoforms *Pt*SUS1 and *Pt*SUS2 (Gerber *et al.*, 2014; An *et al.*, 2014) and may therefore have a specialized function in TW. *Pt*SUS3 transcripts (renamed as *Pt*-SUS2) were also found to be up-regulated in the TW of *P. tomentosa* (Chen *et al.*, 2015).

As expected, several key enzymes of the cellulose biosynthetic machinery were up-regulated in TW, starting at the X2 zone. These included two isoforms of ‘secondary wall’ CesAs (Kumar *et al.*, 2009), *Pt*CesA4 and *Pt*CesA8-B, poplar korrigan *Pt*Cel9A1 (Takahashi *et al.*, 2009; Maloney and Mansfield, 2010) and chitinase-like protein *Pt*GH19A (Aspeborg *et al.*, 2005). Although many previous transcriptomics studies of *Populus* and other hardwoods have suggested the up-regulation of genes involved in cellulose biosynthesis in TW (Paux *et al.*, 2005; Bhandari *et al.*, 2006; Andersson-Gunnerås *et al.*, 2006; Lu *et al.*, 2008; Qiu *et al.*, 2008; Wang *et al.*, 2014*a*), this is, to the best of our knowledge, the first demonstration of their co-up-regulation at the protein level. Recently, Mauriat *et al.* (2015) reported the up-regulation of non-phosphorylated forms of *Pt*CesA8-B and phosphorylated form of *Pt*CesA7-A in TW compared with OW; surprisingly, *Pt*CesA4 (both forms) showed an opposite trend in their study. Clearly, our understanding of the protein complexes involved in TW, OW, and NW formation is still incomplete. Nevertheless, our data support the activation of protein machinery involved in cellulose biosynthesis during the development of cellulose-rich G-layers in TW.

This is further supported by the up-regulation of several α- and β-tubulins in the X4–X6 zones of TW (see Supplementary Fig. S1), since rosette movement depends on the cortical microtubule (MT) network to guide the synthesis and orientation of cellulose microfibril deposition, as well as to provide a way to redirect orientation in response to stimuli (Bringmann *et al.*, 2012). Increased abundance of MTs in developing TW fibers has already been detected by microscopy (Fujita *et al.*, 1974). Some of the α-tubulins have also been reported to be up-regulated in TW at the transcript level (Andersson-Gunnerås *et al.*, 2006; Oakley *et al.*, 2007). Furthermore, two isoforms of microtubule-binding protein, known as translationally controlled tumour protein (TCTP) (Potri.005G024800 and Potri.010G013400) and crucial for microtubule stabilization (Lin *et al.*, 2009), were found to be up-regulated in TW zones X4–X6 (Supplementary Fig. S1). The induction of microtubule subunits and microtubule binding proteins in TW zones X4–X6 reflect the intensification of cellulose biosynthesis at these stages.

UDP-Glc-6-dehydrogenase (UGDH) irreversibly diverts UDP-Glc to UDP-GlcA, which is then fed into pathways that lead to hemicellulose and pectin biosynthesis (Kleczkowski *et al.*, 2011). Two isoforms of UGDH were found to be broadly up-regulated in TW, starting in the C or X1 zones (Fig. 5 and Supplementary Datasets S1 and S3), which suggests that the sugar flux to matrix biosynthesis might be elevated in TW compared with NW. This has not been predicted by earlier transcriptomic analyses (Andersson-Gunnerås *et al.*, 2006). Interestingly, an isoform of UDP-D-glucose/UDP-D-galactose 4 epimerase (UGE) similar to *At*UGE1 was one of most abundant proteins in TW, and up-regulated in zones X4–X6, whereas another isoform similar to *At*UGE2 was down-regulated (Fig. 5 and Supplementary Datasets S1 and S3). UGEs catalysing the conversion between UDP-Glc and UDP-Gal participate in both the biosynthesis and degradation of carbohydrates (Barber *et al.*, 2006). In Arabidopsis secondary xylem, *At*UGE2 and *At*UGE4 provide UDP-Gal for arabinogalactan II biosynthesis whereas *At*UGE1 mainly affects β-1,4-galactan, and, to a lesser extent, arabinogalactan II biosynthesis (Rösti *et al.*, 2007). Our study supports such a role for *Pt*UGE1 in aspen TW since β-1,4-galactan is a major matrix component of G-layers (Mellerowicz and Gorshkova 2012; Gorshkova *et al.*, 2015). Furthermore, UDP-Glc-4,6-dehydratase and UDP-Rha synthase, enzymes that are involved in UDP-Rha biosynthesis, were up-regulated from early stages of xylogenesis in TW (Fig. 5 and Supplementary Datasets S1 and S3), which is consistent with the specific up-regulation of RGI biosynthesis in TW (Gorshkova *et al.*, 2015).

A striking result of our study regarding the nucleotide sugar interconversion pathway was that three isoforms of cytosolic UDP-Xyl synthase (UXS), *Pt*UXS3, *Pt*UXS6, and *Pt*UXS7 (Du *et al.*, 2013), were up-regulated during xylem cell differentiation in TW (Fig. 5 and Supplementary Datasets S1 and S3). The cytosolic isoforms of UXS provide UDP-Xyl for the biosynthesis of xylan and xyloglucan (Ebert *et al.*, 2015; Kuang *et al.*, 2016), and could also contribute UDP-Ara*p* (Seifert, 2004), which is used, for example, in arabinogalactan biosynthesis (Fig. 5 and Supplementary Datasets S1 and S3). Xylan is largely absent in the G-layers of TW but abundant in the S-layers of NW, whereas xyloglucan has been reported to be present in very low amounts in the G-layers and between S- and G-layers of TW but not in the S-layers of either wood type (Nishikubo *et al.*, 2007; Sandquist *et al.*, 2010; Kim and Daniel, 2012; Mellerowicz and Gorshkova, 2012; Gorshkova *et al.*, 2015). Thus, the heightened expression of cytosolic UXSes at late stages of TW formation could contribute to the biosynthesis of xyloglucan, which is thought to be essential for generating tensile stress in G-fibers (Nishikubo *et al.*, 2007; Mellerowicz *et al.*, 2008; Baba *et al.*, 2009; Mellerowicz and Gorshkova, 2012). Other enzymes involved in xyloglucan remodeling, xyloglucan endotransglucosylase (XET) and alpha-xylosidase, were also up-regulated in the stems producing TW (Supplementary Datasets S1 and S3), but only in the phloem. Another role of UXS in TW could be the biosynthesis of arabinogalactan II, which is synthesized as the glycosidic decoration on arabinogalactan proteins (AGPs) and is a major matrix component of G-layers (Gorshkova *et al.*, 2015). Indeed, 13 fasciclin-like arabinogalactan proteins (FLAs), similar to Arabidopsis FLA8, 11, 12 and 17, largely contributed to expression differences found between TW and NW, with most of them highly expressed in TW, especially in zones 4–6 (Fig. 5 and Supplementary Datasets S1 and S3). FLAs have been proposed to contribute to the adhesion between major cell wall components and regulation of cellulose microfibril angle (MacMillan *et al.*, 2010, 2015; Huang *et al.*, 2013). There is copious evidence for the induction of FLAs during TW development from various transcript and proteome investigations of different woody species (Plomion *et al.*, 2000; Lafarguette *et al.*, 2004; Andersson-Gunnerås *et al.*, 2006; Kaku *et al.*, 2009).

The most intriguing finding concerning polysaccharide biosynthetic proteins was the up-regulation of exostosin family proteins homologous to *At*IRX10, *Pt*GT47A1 and 2, in the X4 of TW (Fig. 5 and Supplementary Datasets S1 and S3), which is consistent with previous transcriptomic data (Andersson-Gunnerås, 2006). This result is puzzling, since IRX10 is only known to have xylan xylosyltransferase activity (Urbanowicz *et al.*, 2014; Jensen *et al.*, 2014), and the enzyme is thought to form a xylan synthase complex in the Golgi together with two members of GT43 family, *At*IRX9 and *At*IRX14 (Zeng *et al.*, 2016; Jiang *et al.*, 2016). This function would be expected to be down-regulated in TW since the xylan content of this tissue is reduced and transcripts of all other xylan biosynthetic genes, like *PtGT47C* (*AtFRA8*), *PtGT8B* and *C* (*AtGUX1*), *PtGT8D-1* and *-2* (*AtGAUT12*), and *PtGT8E* and *F* (*AtPARVUS*), were strongly down-regulated in TW (Andersson-Gunnerås *et al.*, 2006; Fagerstedt *et al.*, 2014). Another function proposed for the IRX10 homolog of tobacco *Np*GUT1 is glucuronate transferase activity in the biosynthesis of RGII (Iwai *et al.*, 2002; Iwai *et al.*, 2006). Although this function has not been confirmed in Arabidopsis, this idea is perhaps worth revisiting in aspen TW tissues. The activation of RGII biosynthesis in this tissue could be expected based on the up-regulation of enzymes involved in the biosynthesis of rare sugars that are RGII side chains, apiose, KDO, and L-Gal, which are synthesized by UDP-API/UDP-Xyl synthase 1, 3-deoxy-D-manno-octulosonate-8-phosphate synthase (KDO-8-phosphate synthase), and GDP-Man-3,5-epimerase, respectively (Fig. 5 and Supplementary Datasets S1 and S3).

### Lignin biosynthetic proteins

**Fig. 6.**
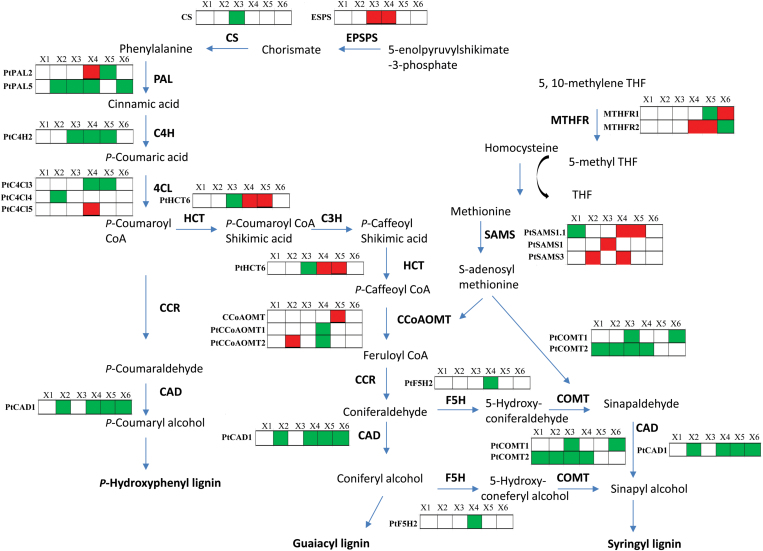
Differentially expressed enzymes involved in lignin biosynthesis pathways leading to monolignols in xylems zones X1–X6 of tension wood development, as compared with normal wood formation. Green, up-regulation; red, down-regulation. The proteins include the following: 5-enolpyruvylshikimate-3-phosphate synthase (EPSPS; Potri.002G146400); chorismate synthase (CS; Potri.010G221600); two isoforms of phenylalanine ammonia-lyase, *Pt*PAL2 and 5 (Potri.008G038200, Potri.010G224100); ferulic acid 5-hydroxylase *Pt*F5H2 (Potri.007G016400); two isoforms of *O*-methyltransferase, *Pt*COMT1 and 2 (Potri.015G003100, Potri.012G006400); three isoforms of 4-coumarate CoA ligase, *Pt*4CL3, 4 and 5 (Potri.001G036900, Potri.019G049500, Potri.003G188500); cinnamate-4-hydroxylase (C4H2; Potri.019G130700); three isoforms of caffeoyl-CoA *O*-methyltransferase, *Pt*CCoAOMT1 and 2 (Potri.009G099800, Potri.001G304800) and CCoAOMT (Potri.008G136600), with its closest homolog to *Pt*CoAOMT4 (Shi *et al.*, 2010), p-hydroxycinnamoyl-CoA: shikimate/quinate *p*-hydroxycinnamoyltransferase *Pt*HCT6 (Potri.003G183900); alcohol dehydrogenase *Pt*CAD1 (Potri.009G095800); methylenetetrahydrofolate reductase (MTHFR; Potri.007G147300); and three isoforms of *S*-adenosyl-L-methionine synthase, *Pt*SAMS1, 1.1 and 3 (Potri.013G004100, Potri.014G114700, Potri.010G153500).

Enzymes involved in lignification were mainly detected in the X2-X6 samples of both NW and TW (Fig. 6 and Supplementary Dataset S1 and S3). This profile follows the theoretical requirements for lignin monomers, since lignification starts after the initiation of the secondary cell wall layer and may proceed even after cell death (Boerjan *et al.*, 2003; Shi *et al.*, 2010; Pesquet *et al.*, 2013; Obudulu *et al.*, 2016). An isoform in the shikimate pathway that guides the flow of carbon from sugar metabolism to phenylalanine biosynthesis, 5-enolpyruvylshikimate-3-phosphate synthase (EPSPS), was down-regulated in TW (Fig. 6), which is in agreement with previously reported down-regulation of *EPSPS* transcripts in the TW of *Betula platyphylla* (Wang *et al.*, 2014*a*) and *Populus tremula* (Andersson-Gunnerås *et al.*, 2006). Several enzymes from the phenylpropanoid pathway were down-regulated in TW, especially in X3, X4 or X5, for example, an isoform of phenylalanine ammonia-lyase, *Pt*PAL2; 4-coumarate CoA ligase, *Pt*4CL5; *p*-hydroxycinnamoyl-CoA-quinate shikimate *p*-hydroxycinnamoyltransferase, *Pt*HCT6; and caffeoyl-CoA *O*-methyltransferase, *Pt*CCoAOMT and *Pt*CCoAOMT2 (Fig. 6). However, many other enzymes from this pathway were also up-regulated in X zones of TW, including *Pt*PAL5, cinnamate-4-hydroxylase *Pt*C4H2, *Pt*4CL3 and 4, cinnamyl alcohol dehydrogenase *Pt*CAD1, ferulic acid 5-hydroxylase *Pt*F5H2, and *O*-methyltransferase *Pt*COMT1 and 2 (Fig. 6). *Pt*4CL5 have been suggested to form a heterotetrameric protein complex with *Pt*CL3 and appears also to have a regulatory role in *Populus* (Chen *et al.*, 2014).

Unfortunately, there are few proteomic studies regarding the regulation of lignin biosynthesis during TW formation. However, the proteins phenylalanine ammonia lyase, cinnamoyl-CoA reductase, caffeoyl-CoA *O*-methyltransferase and cinnamate 4-hydroxylase, which participate in lignin biosynthesis, are generally found to be down-regulated (e.g. Mauriat *et al.*, 2015), and transcript data show a reduction in most of the lignin biosynthetic genes in the developing xylem region of TW in *Populus* and *Betula platyphylla* (Andersson-Gunnerås *et al.*, 2006; Wang *et al.*, 2014*a*; Chen *et al.*, 2015). This reduction in lignin biosynthetic proteins might be expected since it has been documented that TW has lower lignin content than NW (Fagerstedt *et al.*, 2014). However, the lignification of the middle lamella (CML) and the S1 and S2 layers of the cell wall may continue well after formation of these layers (Yoshinaga *et al.*, 2012), and this process may last longer in TW due to its longer overall differentiation. Furthermore, in our experiment, field grown aspen trees were used, which also might have an impact on results compared with results with greenhouse grown trees.

A pathway related to lignin biosynthesis, which involves methylenetetrahydrofolate reductase (MTHFR) and three *S*-adenosyl-L-methionine synthases (SAMS; *Pt*SAMS1, *Pt*SAMS1.1, *Pt*SAMS3), was observed to be down-regulated in X zones of TW (Fig. 6 and Supplementary Dataset S1 and S3). MTHR is essential in methionine biosynthesis and SAMS catalyses the synthesis of *S*-adenosyl-L-methionine (SAM) from L-methionine and ATP. SAM acts as a general methyl-group donor in several transmethylation reactions (Boerjan *et al.*, 1994), including transmethylations of structural constituents of the cell wall including several reactions that occur in the biosynthesis of lignin (Campbell and Sederoff, 1996). In accordance with our findings, SAMS1 was one of the most abundant transcripts in a cDNA library of developing *Populus* NW examined by Andersson-Gunnerås *et al.* (2006). Furthermore, in a proteomic study of compression wood, which has high lignin content (Timell, 1986), in maritime pine (Plomion *et al.*, 2000), SAMS was found to be up-regulated. The MTHFR mutant in maize (bm2) (Tang *et al.*, 2014), which has lower concentrations of SAM, and the *At*SAMS3 mutant in Arabidopsis (similar to *Pt*SAMS1) (Shen *et al.*, 2002) have both been shown to lead to a decrease in the accumulation of G- and S-lignin. The observed reductions of SAMS and MTFHR isoforms in TW probably affect SAM metabolism in TW tissue, and could thus be important regulatory factors in lignin biosynthesis, shown by previously reported lignin reductions in this tissue. However, the previously reported *in vitro* kinetic metabolic-flux models (Wang *et al.*, 2014*b*) for monolignols and enzymes involved in lignin biosynthesis must also be performed in *in vivo* systems to pinpoint the specific roles of various isoforms in TW development.

## Conclusions

The application of proteomic analysis to samples representing various stages of *Populus* TW and NW formation provided valuable information that had not been revealed by earlier transcriptome studies. Our research also identified many protein isoforms that are potentially important in the biosynthesis of the different cell layers during TW formation. We have proposed functions for some of the identified proteins, which may be good targets for functional and high resolution targeted proteomics analyses that aim to explain the cellular mechanisms underlying xylogenesis and/or TW formation.

## Supplementary data

Supplementary data are available at *JXB* online.

Dataset S1. List of proteins quantified and compared in the pairwise transition models between tension wood normal wood.

Dataset S2. Statistical enrichment analysis of GO terms (REVIGO) corresponding to proteins with higher expression in tension wood compared with normal wood, and based on unique proteins from Datset S1.

Dataset S3. Lignocellulosic regulated proteins in *Populus* tension wood compared with normal wood.

Figure S1. Signaling, RedOx and Tubulin regulated proteins in *Populus* tension wood compared with normal wood.

## Data deposition

The mass spectrometry proteomics data have been deposited to the ProteomeXchange Consortium via the PRIDE (Vizcaíno *et al.*, 2016) partner repository with the dataset identifier PXD005715.

## Supplementary Material

Supplementary Dataset S1

Supplementary Dataset S2

Supplementary Dataset S3

Supplementary Figure S1
