# Supplementary material for: Protein expression in tension wood formation monitored at high tissue resolution in Populus
Source: J Exp Bot. 2017 Jun 13;68(13):3405–17. doi: 10.1093/jxb/erx186 (PMC5853651; doi:10.1093/jxb/erx186)

Supplementary Fig. S1. Signaling, RedOx and Tubulin regulated proteins in *Populus tremula* tension wood (TW) compared with normal wood (NW). Sections: cambium (C), phloem (P), expansion (E) and xylem (X1-X6). Green represents higher expression in TW and red represents lower expression in TW. In brackets: number of unique quantified peptides (U), and non-unique (NU) peptides. Classification of proteins in brackets either as *Populus* (Pt) or *Arabidopsis* (At) annotated genes.

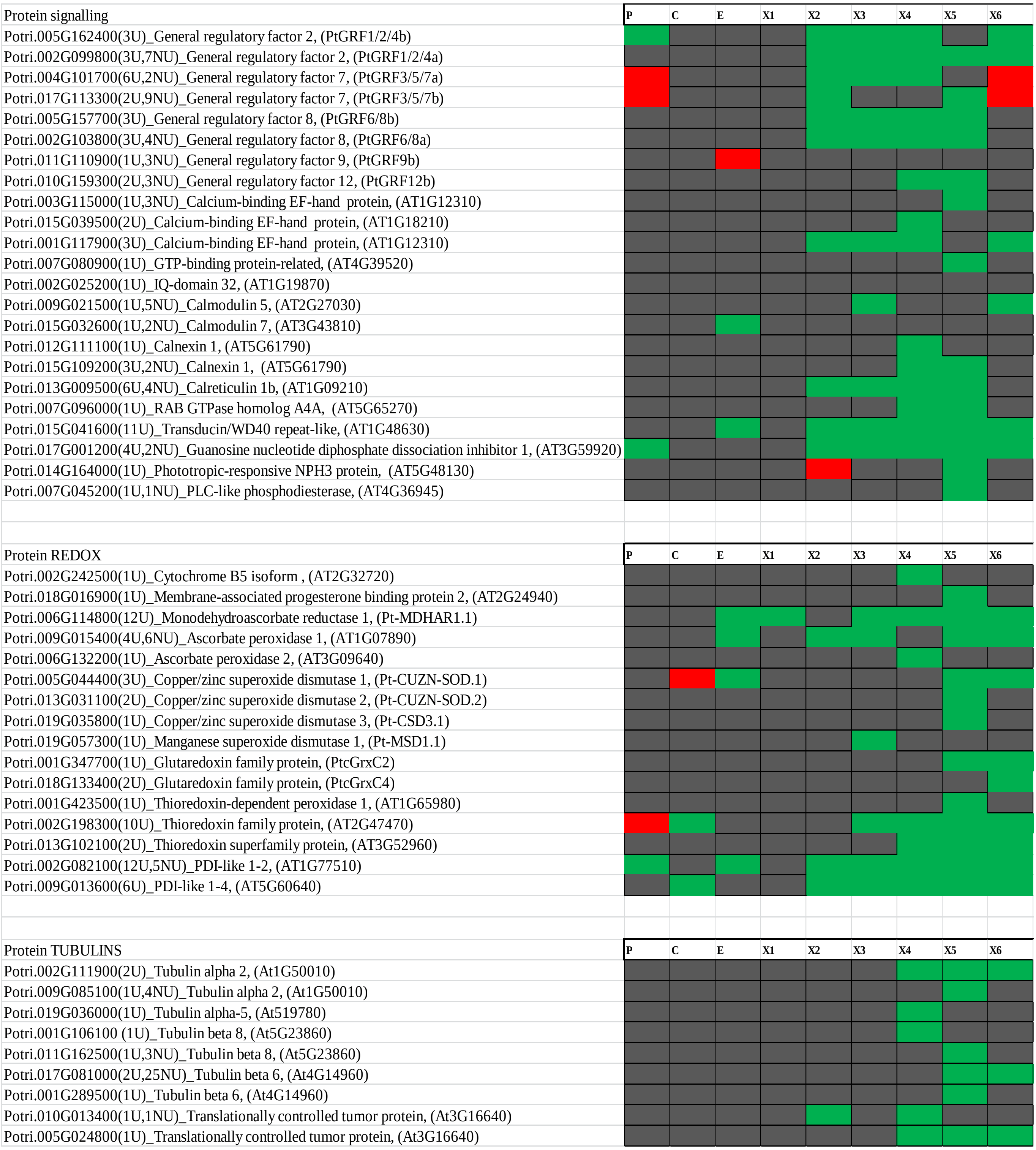

Supplement: Supplementary Figure S1 [file erx186_suppl_supplementary_figure_s1.pdf]
